# The relationship of mandibular radiomorphometric indices to skeletal age, chronological age and skeletal malocclusion type

**DOI:** 10.4317/jced.53819

**Published:** 2017-08-01

**Authors:** Ali Tayebi, Maryam Tofangchiha, Mahsa-Arian Fard, Armin Gosili

**Affiliations:** 1 Assistant Professor of Orthodontics, Department of Orthodontics, Qazvin Medical University, Qazvin, Iran; 2 Associate Professor of Oral and Maxillofacial Radiology, Department of Oral and Maxillofacial Radiology, Qazvin Medical University, Qazvin, Iran; 3 Dentist, Private Practice, Tabriz, Iran; 4 Post Graduate Student in Orthodontics, Department of Orthodontics, Qazvin Medical University, Qazvin, Iran

## Abstract

**Background:**

The present study was performed with the following aims: (1) to assess the relationship between skeletal age, measured using the cervical vertebral maturity (CVM) method, and chronological age; (2) to determine the correlation of skeletal and chronological age to the cortical thickness of the lower border of the mandible using the linear radiomorphometric; and (3) to explore the relationship between these indices and skeletal malocclusion type.

**Material and Methods:**

The data were collected from the records of 180 patients, including 57 males (31.7%) and 123 females (68.3%). The data were based on the panoramic and lateral cephalograms of each patient. The CVM stages were determined on the basis of the patients’ lateral cephalograms. Three radiomorphometric indices were measured: AI, MI and GI. The patients were divided up into three groups of skeletal malocclusion: Class I, II, and III. For all the tests, statistical significance was set at *P*<0.05.

**Results:**

The relationship between chronological age and skeletal age was 0.496. Furthermore, with an increase in chronological and skeletal age, the cortical thickness of the lower border of the mandible and consequently the radiomorphometric indices increase, except for the GI (*P* > 0.05). Lastly, the relationship between GI and skeletal malocclusion type proved significant.

**Conclusions:**

AI and MI were found to increase significantly with increasing age, so the assessment of mandibular radiomorphometric indices could be clinically useful in estimating of the growth and maturation of the mandible.

** Key words:**Orthodontics, Radiomorphometric indices, Skeletal age, Skeletal malocclusion.

## Introduction

Determination of maturity and growth potential remaining in patients plays a vital role in providing orthodontic treatments. Hence, in some cases, especially in functional and orthopedic treatment of skeletal abnormalities, the entire treatment plan depends on the patient’s stage of development and the amount of growth. Maturity has an important role in the treatment prognosis of skeletal abnormalities (1,2).

Chronological age is not a reliable indicator in determining the amount of bone development (3). Thus, some other indicators have been propounded. Furthermore, methods such as weight gain, menstruation cycle onset, or voice changes have little validity in determining the rate of growth spurt (4,5). Therefore, the use of radiography for estimating the rate of skeletal maturity is considered. Another efficient and reliable method is the use of hand-wrist radiographs (6-8). This method has advantages, but one of the concerns is the need for additional radiographs, which means more radiation being received by the patient. Evaluating the cervical vertebrae visible on lateral cephalometric radiographs is another technique used for determining the skeletal age of the patients, thereby decreasing the patient’s radiation exposure (3,4,8-11).

**Figure 1:**
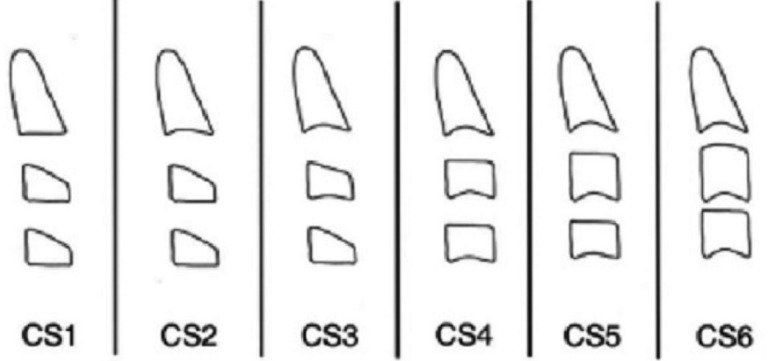
The Baccetti technique used to determine the morphological changes in the cervical vertebrae (10).

Baccetti *et al.* recommended the use of the cervical vertebral maturation (CVM) method as a biological indicator, both for mandibular and somatic maturation (4,10). This method analyzes the morphology of the second (C2), third (C3), and fourth (C4) cervical vertebrae, and the patient is placed in one of the five stages: CVMS I to CVMS V. The inferior vertebral borders are flat when least mature, but with maturation they become concave. The concavities become more obvious as maturation occurs (Fig. 1) (10).

The cervical vertebrae are readily visible on lateral cephalometric radiographs, which are regularly recorded for most orthodontic patients (10). On the other hand, one of the most common radiographic methods is panoramic radiograph, which is often used in diagnosis and treatment planning. In consequence, evaluation can be made of a large number of quantitative and qualitative measurements of mandibular bone obtained from panoramic radiographs (12,13), including densitometry and morphometry (14). Complete understanding of bone evolution and maturation is necessary, and valid indices are useful in this regard.

It is easy to observe the cortical thickness of the inferior border of the mandible on each panoramic radiograph and to assess mandibular morphometric indices for the purpose of finding out about bone status (13,15).

In the literature, there are a large number of studies using panoramic radiography to explain the effects of age, sex, and body mass index (BMI) on the thickness of the mandibular cortex (12,13,15-17). However, all the patients examined in these studies were elderly people. There is only one study, to the best of our knowledge, which uses radiomorphometric indices for evaluating the growth and development of young people’s mandible (14). On the other hand, radiographic assessment of bone quantity significantly contributes to planning further dental treatment orthodontics included.

The purpose of the present study is three-fold: first, to determine the relationship between skeletal age, measured through the CVM method, and chronological age; second, to assess the correlation of skeletal and chronological age to the cortical thickness of the lower border of the mandible, measured using the linear radiomorphometric indices of mental index (MI), gonial index (GI), and antegonial index (AI); and third, to evaluate the relationship between skeletal malocclusion type and the aforementioned indices in young male and female patients.

## Material and Methods

This research is a descriptive-retrospective study. Simple sampling was performed, with the samples being selected from patients at the Department of Orthodontics, Faculty of Dental Medicine, Qazvin University of Medical Science. A total of 180 patients who had received orthodontic treatment were evaluated in this study. These patients were all eligible and their records were available in the Archives Department of the Faculty. All were Iranian and had no history of known diseases affecting general body growth such as hormonal diseases. They did not have orthodontic treatment prior to the study and did not use medications such as estrogen, progesterone, and steroids. Of all the patients, 57 (31.6%) were males and 123 (68.3%) were females. Chronological ages of the participants ranged from 7 to 13 years old, with a mean of 9.77 for males and 9.46 for females. The overall mean chronological age was 9.56.

All lateral cephalograms and panoramic radiographs were appropriate in terms of visual and technical quality (i.e., contrast and density) and were prepared under the supervision of a maxillofacial radiologist.

Exclusion criteria were the presence of pathological lesions associated with teeth or lower border of the mandible in panoramic radiographs, fuzziness of C2, C3, and C4 vertebrae in lateral cephalometric radiographs, unknown date of radiographs taken, radiographs without diagnostic value, images containing technical errors, and problems like severe malnutrition, and uncertain and ambiguous date of birth.

-Assessment of skeletal malocclusion

radiographic observations based on cephalometric analysis were used to assess types of skeletal malocclusion. Patients were divided up into three groups in terms of skeletal malocclusion: Class I, II, and III. Cephalometric analysis indicated that 39.4% of the patients had Class I malocclusion, 53.3% Class II, and 7.2% were Class III ( Table 1).

**Table 1:**
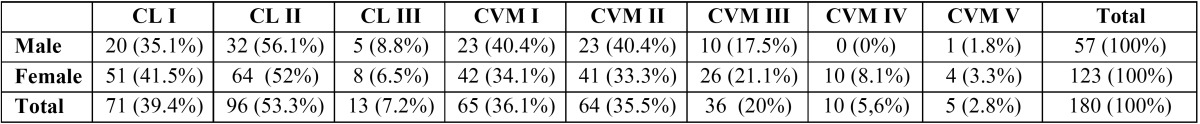
The distribution of the skeletal malocclusion and CVM stages of the participants as number and percentage.

-Assessment of skeletal development

Skeletal development of the patients was determined by an orthodontist from lateral cephalometric radiographs based on an evaluation of the cervical vertebrae according to the method described by Baccetti *et al.* The distribution of the CVM stages of the participating patients is given in  Table 1.

-Measurements of radiomorphometric indices

The mandibular radiomorphometric indices of GI, AI, and MI were measured on panoramic radiographs. A tracing paper (Ortho Organizer type paper) was attached to all confirmed panoramic radiographs. Tracing was performed by a trained student. MI, GI, and AI were determined by a maxillofacial radiologist and were then measured using a digital Guanglu type caliper (Guilin Guanglu Measuring Instrument Co., Nanning, Guangxi, China). The indices at issue were observed bilaterally and the measurements were performed on both sides of panoramic radiographs. However, in the cases where the indices on one side were not clear, only the indices of the other side were used in measurements. The data of each patient were recorded on separate forms.

**Figure 2:**
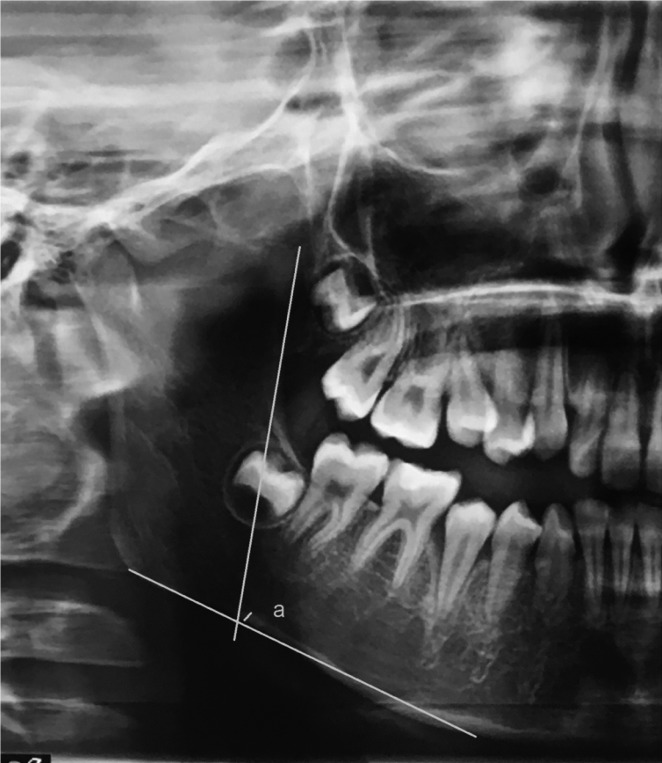
Antegonial Index (a); (Length of “a” in mm).

Antegonial Index (AI): the most recent quantitative index, first expressed by Ledgerton *et al.* AI consists of the lower cortical thickness in the area of antegonial at a point along the line of the anterior ascending ramus, pulling down and confluence with the inferior cortex. AI (Fig. 2) is the measurement of the mandibular cortical thickness measured on the line perpendicular to the mandibular cortex at the intersection with the line tangent to the anterior border of the ramus (normal value > 3.2 mm) (19).

**Figure 3:**
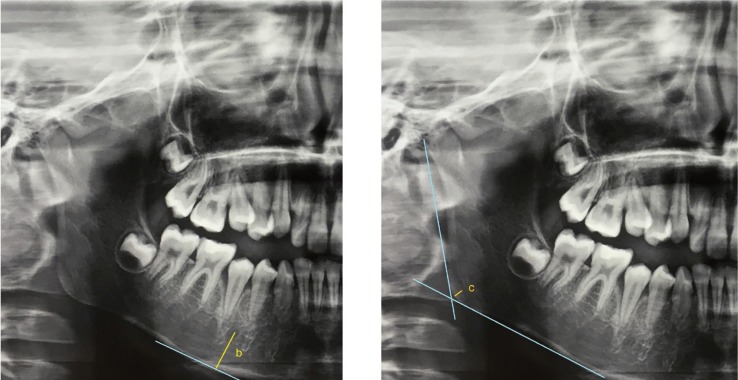
Mental Index (b); and Gonial Index (c); (Length of “b and c” in mm).

Mental index (MI): mandibular cortical width at the mental region. It is one of the quantitative indices used to assess bone quality (Fig. 3). MI was measured based on the appearance of the cortical border of the mandible distal to the mental foramen. It is the measurement of the mandibular cortical thickness on the line perpendicular to the bottom of the mandible at the middle of the mental foramen (normal value > 3.1 mm) (14,17).

Gonial index (GI): cortical width in the gonial area. GI is the measurement of the mandibular cortical thickness measured on the bisector of the angle between the lines tangent to the posterior border of the ramus of the mandible and the bottom of the mandible (normal value > 1.2 mm) (17,18).

Paired-samples t-tests revealed no statistically signiﬁcant difference for mandibular radiomorphometric indices between the left and right side of the mandible and also between males and females (*P* > 0.05). Thus, the average of the two side of the mandible were used in all further statistical analyses. The minimum, maximum, mean, and standard deviation (SD) values of the three radiomorphometric indices under study (i.e., GI, AI, and MI) measured on the left and right side and for three skeletal malocclusion types are presented in  Table 2.

**Table 2:**
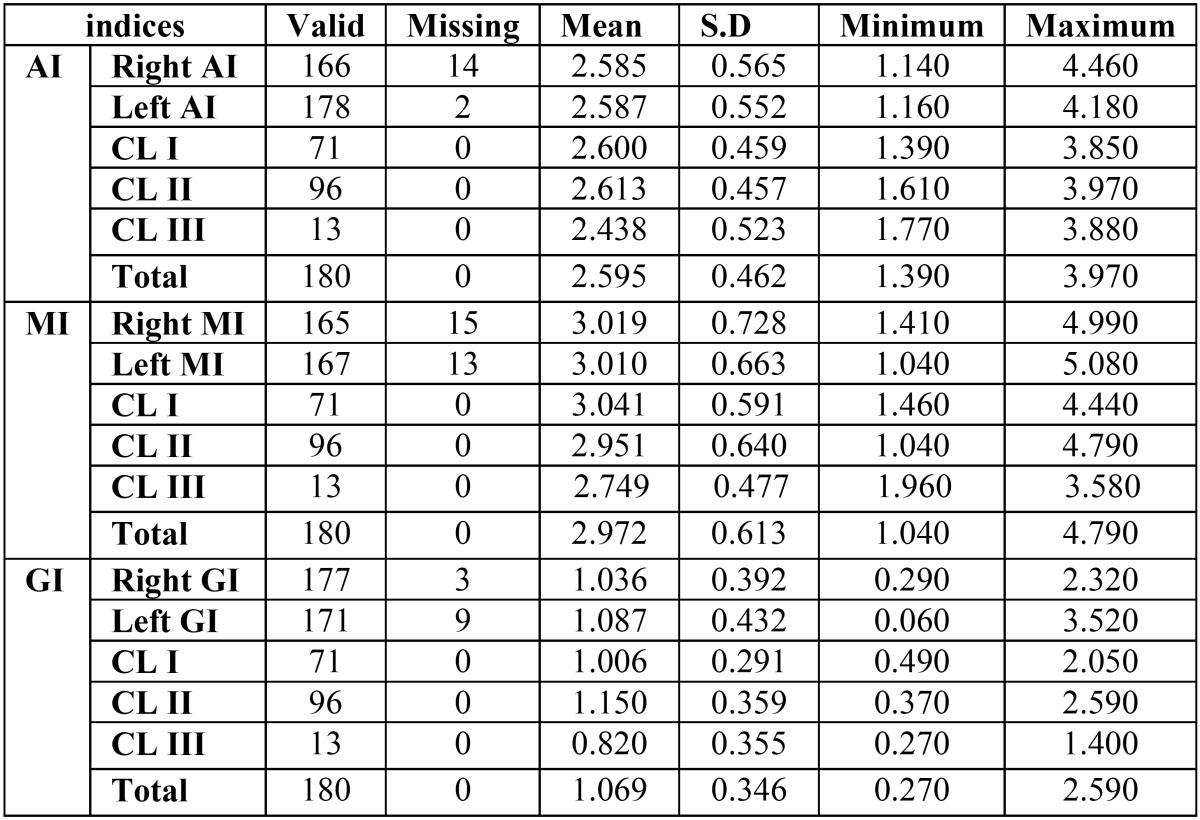
Number of valid and missing measurements, Mean, SD, minimum and maximum values of mandibular radiomorphometric indices for left and right sides of mandible and for three skeletal malocclusion type in millimeters.

-Data analysis

Once the data were gathered, they were analyzed using SPSS 21 (IBM Corporation, NY, USA, 2012). The correlation between chronological age and skeletal age as well as the correlation of chronological and skeletal age to radiomorphometric indices were determined using Pearson product-moment correlation coefficient. Additionally, one-way ANOVA and the Tukey tests were used to explore the relationship between skeletal malocclusion type and the mandibular radiomorphometric indices. We used an alpha level (Type I error) of 0.05 in this research.

-Ethical considerations

This study is ethically acceptable given that it was conducted using archived records of the patients. Moreover, there was no need to take additional radiographs and unnecessary radiation exposure.

## Results

A significant relationship was found between skeletal and chronological age such that with an increase in chronological age, skeletal age of the patients’ progresses from CVM1 to CVM5. Pearson correlation coefficient between chronological age and skeletal age was 0.496.

It was also found that radiomorphometric indices of mandibular cortex are influenced by skeletal age. AI and MI get thicker with skeletal age ( Table 3). However, GI had no significant relation with skeletal age (*P* > 0.05).

**Table 3:**
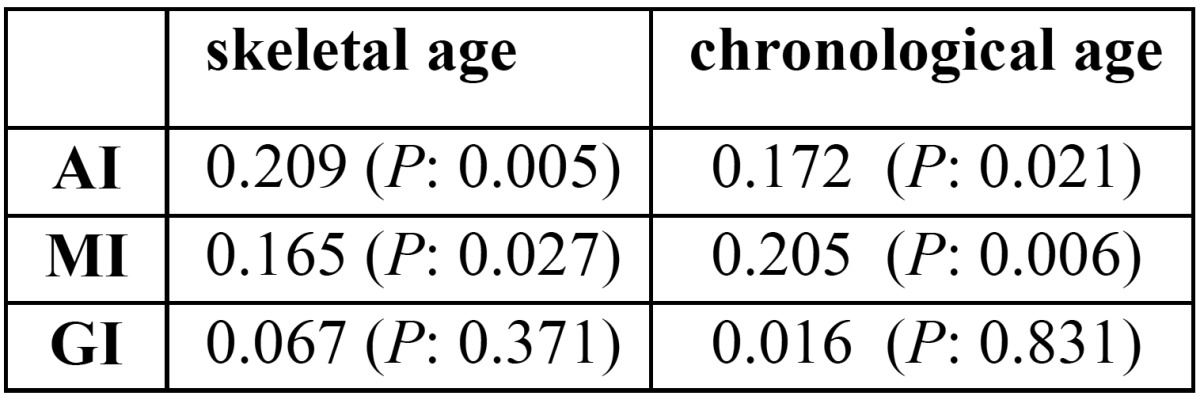
Correlation of skeletal and chronological age to mandibular radiomorphometric indices.

Radiomorphometric indices of mandibular cortex were also found to be influenced by chronological age. As  Table 3 shows, there was a significant increase in AI and MI values when the chronological age increased. although GI had no significant relation with chronological age (*P* > 0.05).

As for the relationship between skeletal malocclusion type and the mandibular radiomorphometric indices, the mean AI values were almost the same for all groups, with slightly higher values for Class II than Class III. The mean MI values were the highest in the Class I, a little lower in Class II and the lowest in Class III group. Statistically significant difference was found only for the GI. Specifically, according to Tukey analysis of data, only the difference between Class II and Class III malocclusion for GI index was significant ( Table 4). the highest mean GI values were observed in Class II malocclusion (1.150 mm), and the lowest in Class III malocclusion (0.820 mm), The GI mean value was 1.006 mm in Class I malocclusion.

**Table 4:**
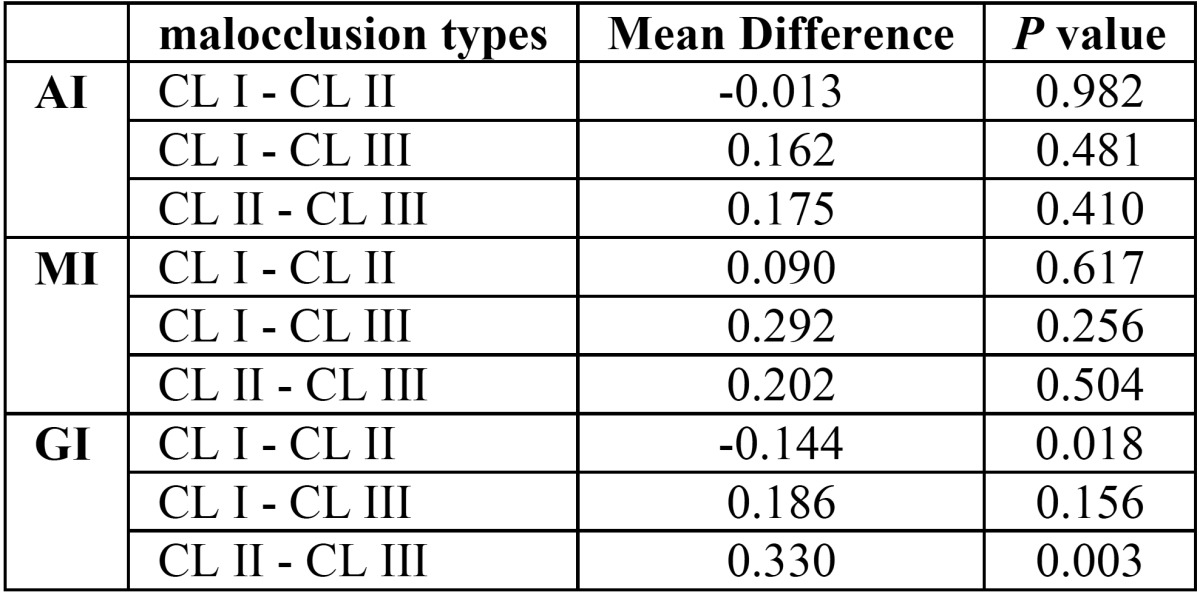
Correlation of skeletal and chronological age to mandibular radiomorphometric indices.

## Discussion

The cervical vertebrae are readily observable on lateral cephalometric radiographs, which are typically recorded for a vast majority of orthodontic patients. Dentofacial structures are known to have different growth rates during growth and development. This is fundamental, particularly in the treatment and stability of skeletal anomalies (19). The fact that cervical vertebrae exhibit varying anatomical shapes and features during different maturation stages can be beneficial in the assessment of skeletal maturation using CVM (4,10,20).

The present study observed a significant relationship between skeletal age and chronological age, but this correlation was low (r = 0.496). This finding is consistent with the results reported in Safavi *et al.*, who found a relatively low (r = 0.620) correlation coefficient between CVM stages and chronological age (21). However, Alkhal *et al.* observed relatively high correlations (males: r = 0.757; females: r = 0.787) in this respect (22). Likewise, Uysal *et al.* and Soegiharto *et al.*, observed relatively high correlation between chronological age and CVM stages (23,24).

The variety observed in the coefficient values can be attributed to the fact that maximum craniofacial growth speed is a complex factor and is strongly influenced by genetics (21,25) other reason for this, could be the small sample size.

Another finding of the present study was that radiomorphometric indices increase as chronological and skeletal age increase. an increase occurs in mandibular cortical thickness between the ages of 7 and 13. More specifically, AI and MI were found to increase significantly with skeletal and chronological age. There is only one study (Zlatarić, 2006), to the best of our knowledge, which uses radiomorphometric indices for evaluating the growth and development of the mandible in young adolescents. The findings of the two studies in this relation are similar. Zlatarić (2006) ascribed the increase in mandibular cortical thickness to the increased bite force coincident with maturity (14).

The low thickness of the gonial area could be held responsible for the lack of a significant correlation of GI to chronological and skeletal age. Another cause of this insignificant relationship can be the small sample size.

Finally, a significant relationship was found between GI and skeletal malocclusion type. More particularly, the highest and lowest values of GI were observed in Class II and Class III malocclusion types, respectively. This can be because the gonial area of the mandible is the region for masseter muscle attachment, and It has been demonstrated that the activity of masticatory muscles, especially the masseter, varies according to the type of malocclusion (26).

Clinical research has assessed the role of masticatory muscles on normal and abnormal growth of facial structures (27). Muscle activity is reﬂected by the varying degrees of force applied to the bone at muscular attachment sites (28). Thus, the increase or decrease in mandibular cortical thickness emanates from the application of different degrees of force to the cortical bone.

This finding contradicts the study performed by Zlatarić (2006), in which patients with Class III malocclusion exhibited the highest value of GI. This discrepancy could be due to race, type of diet, or other differences between the two studies.

## Conclusions

Based on the results of the present study:

1) A significant relationship was found between skeletal and chronological age.

2) Radiomorphometric indices increase as chronological and skeletal age increase, indicate an increase in mandibular cortical thickness between the ages of 7 to 13 years.

3) AI and MI were found to increase significantly with increasing skeletal and chronological age.

4) No significant differences were found between the right and the left side of the mandible and between genders for AI, MI and GI (*p*>0.05).

5) Mandibular cortical thickness in Gonial area (GI) based on the type of malocclusion was different, and demonstrated the hig-hest value in Class II malocclusion.

Finally, assessment of the mandibular radiomorphometric indices could be clinically useful in estimating of the growth and maturation of the mandible.
